# Interventional Physiatry in the Complex Regional Pain Syndrome of the Upper Limb Following Herpes Zoster

**DOI:** 10.7759/cureus.74081

**Published:** 2024-11-20

**Authors:** Raktim Swarnakar, Anoovab Saha, Ankit Sarkar, Soumyadipta Ghosh, Pankaj K Mandal

**Affiliations:** 1 Physical Medicine and Rehabilitation, RG Kar Medical College and Hospital, Kolkata, IND

**Keywords:** complex regional pain syndrome, pain, physiatry, physical medicine and rehabilitation, postherpetic neuralgia, herpes zoster

## Abstract

A 44-year-old male patient experienced persistent radiating pain from the elbow to the hand following herpes zoster vesicular eruptions three months earlier. His examination met the Budapest Clinical Criteria for Complex Regional Pain Syndrome (CRPS), revealing sensory, motor, vasomotor, and sudomotor signs and symptoms. Despite conservative treatments, the pain persisted. The patient received an ulnar and median nerve block using a mixture of 10 mg methylprednisolone and 2% lignocaine (30 mg). The ulnar nerve block was administered at two sites: Guyon’s canal and one fingerbreadth distal to the ulnar styloid for the cutaneous branch. The median nerve block was performed at the carpal tunnel. Seven days post-intervention, the patient reported significant pain relief, and by day 15, pain remission was complete. The patient's range of motion improved and the swelling decreased notably. Post-herpetic CRPS in the upper limb is an uncommon complication of herpes zoster. Previous studies have shown “CRPS-like” symptoms following herpes zoster, and this case highlights the value of a multimodal treatment approach that combines interventional techniques with physical therapy. This case illustrates the rarity of post-herpetic CRPS and the effectiveness of peripheral nerve blocks, medication, and exercise in achieving significant pain relief and functional recovery.

## Introduction

Herpes zoster (shingles) is a viral infection caused by the reactivation of the varicella-zoster virus, often leading to painful vesicular eruptions along the affected dermatome. While the acute symptoms typically resolve within a few weeks, some patients may develop persistent pain, which can significantly impair the function and quality of life [1]. This persistent pain, often described as burning or radiating, may progress into postherpetic neuralgia. On the other hand, Complex Regional Pain Syndrome (CRPS) is a debilitating condition characterized by sensory, motor, autonomic, and trophic changes affecting the extremities, with symptoms that can persist long after the initial injury or infection has resolved [2]. The diagnosis of CRPS relies on clinical criteria, such as the Budapest Clinical Criteria, which include sensory changes (e.g., hyperalgesia or allodynia), motor dysfunction (e.g., weakness, tremors), and autonomic manifestations (e.g., changes in skin temperature, color, or sweating) [3]. Despite the use of conservative treatment options, such as analgesics, physical therapy, and nerve blocks, the pain can remain refractory in some patients, requiring more targeted interventions.

A set of interventions in the field of Physical Medicine and Rehabilitation (PMR) aimed at improving functional outcomes is referred to as interventional physiatry. This approach includes evidence-based exercises, physical modalities, medications, orthotic and prosthetic management, injection procedures, as well as occupational and vocational rehabilitation [4]. In this case, we have highlighted the uncommon presentation of CRPS following herpes zoster, along with an interventional physiatry approach to treatment.

## Case presentation

**Figure 1:**
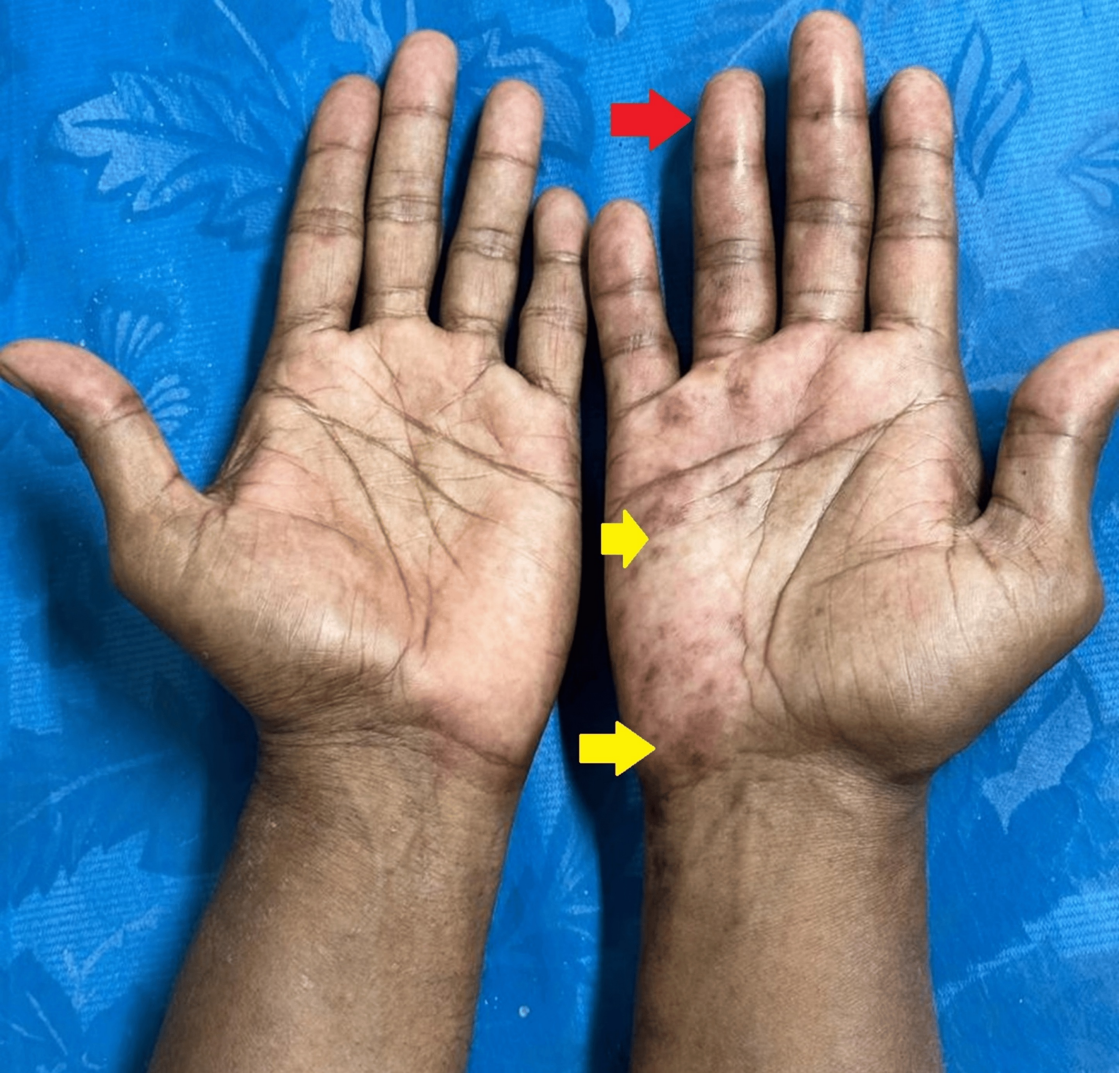
Postherpetic complex regional pain syndrome of right hand (before intervention) Swelling of the finger (red arrow); postherpetic lesions (yellow arrows)

A 44-year-old male patient presented to the PMR outpatient department with persistent radiating pain from the right elbow to the hand. He had a history of herpes zoster vesicular eruptions three months earlier, affecting the ulnar distribution of the right hand and forearm. There was no history of trauma. Due to the pain, he experienced significant disability in performing the Activities of Daily Living (ADLs) because of impaired hand function (Barthel index: 65 out of 100) [5]. On examination, the patient met the Budapest Clinical Criteria for CRPS, with sensory (decreased sensation), motor (developing partial claw hand deformity with weakness), vasomotor (temperature and skin color asymmetry compared to the left hand), and sudomotor (swelling/edema) signs and symptoms (Figure 1). Additionally, his right wrist flexion (10 degrees) and extension (10 degrees) were restricted. 

All relevant blood tests were within normal limits, including the total leucocyte count, which was 10,000 cells per microliter (reference: 4,000-11,000 cells/µL), and serum C-reactive protein, which was 0.1 milligrams per deciliter (reference: <1.0 mg/dL). Serological tests for hepatitis and human immunodeficiency virus were negative. He had been taking gabapentin (1200 mg/day), pregabalin (150 mg/day), and occasionally opioids (tramadol 200 mg/day) over the past three months. He took these medications at varying doses and at different times, but he was unable to adhere to higher doses due to side effects. Pain reduction was minimal following medications [Visual analogue scale (VAS) score of 8 to 7]. Despite conservative treatments, the pain persisted.

**Figure 2:**
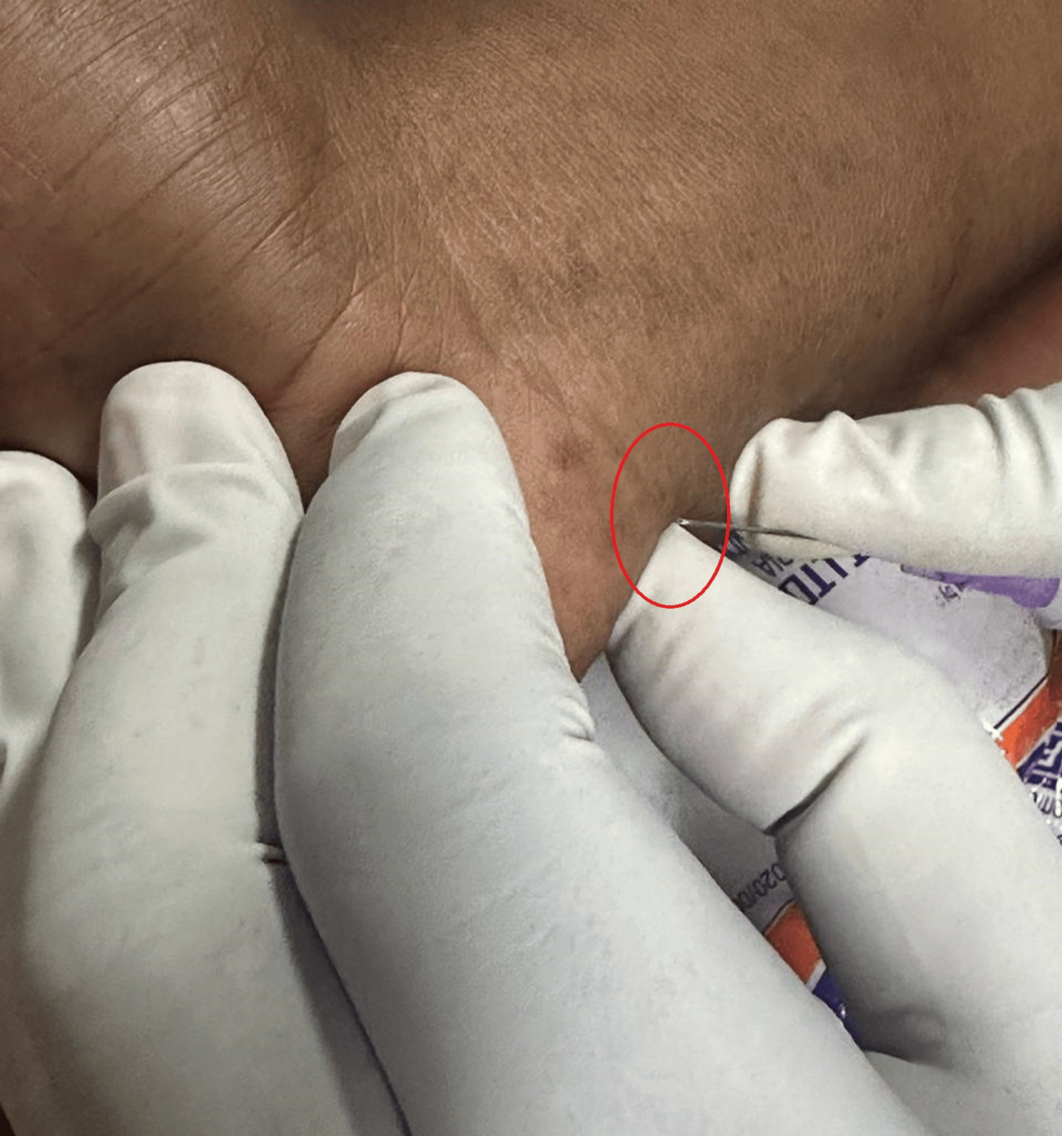
Injection near Guyon's canal Needle tip near Guyon's canal (red circle)

In the rehabilitation setting, the patient was prescribed a regimen of exercises, including range-of-motion exercises for the right wrist, fingers, elbow, and shoulder, as well as stretching exercises for wrist flexors, finger flexors, and strengthening exercises for wrist extensors, flexors, and intrinsic hand muscles [6]. The range-of-motion exercises were initiated first, followed by the gradual introduction of the other exercises. Only gabapentin (400 mg/day) was prescribed. The patient also received ulnar and median nerve blocks using a mixture of 10 mg methylprednisolone and 2% lignocaine (30 mg), administered under strict aseptic precautions [7]. The ulnar nerve block was performed at two sites: Guyon’s canal and one fingerbreadth distal to the ulnar styloid for the cutaneous branch (Figure 2).

**Figure 3:**
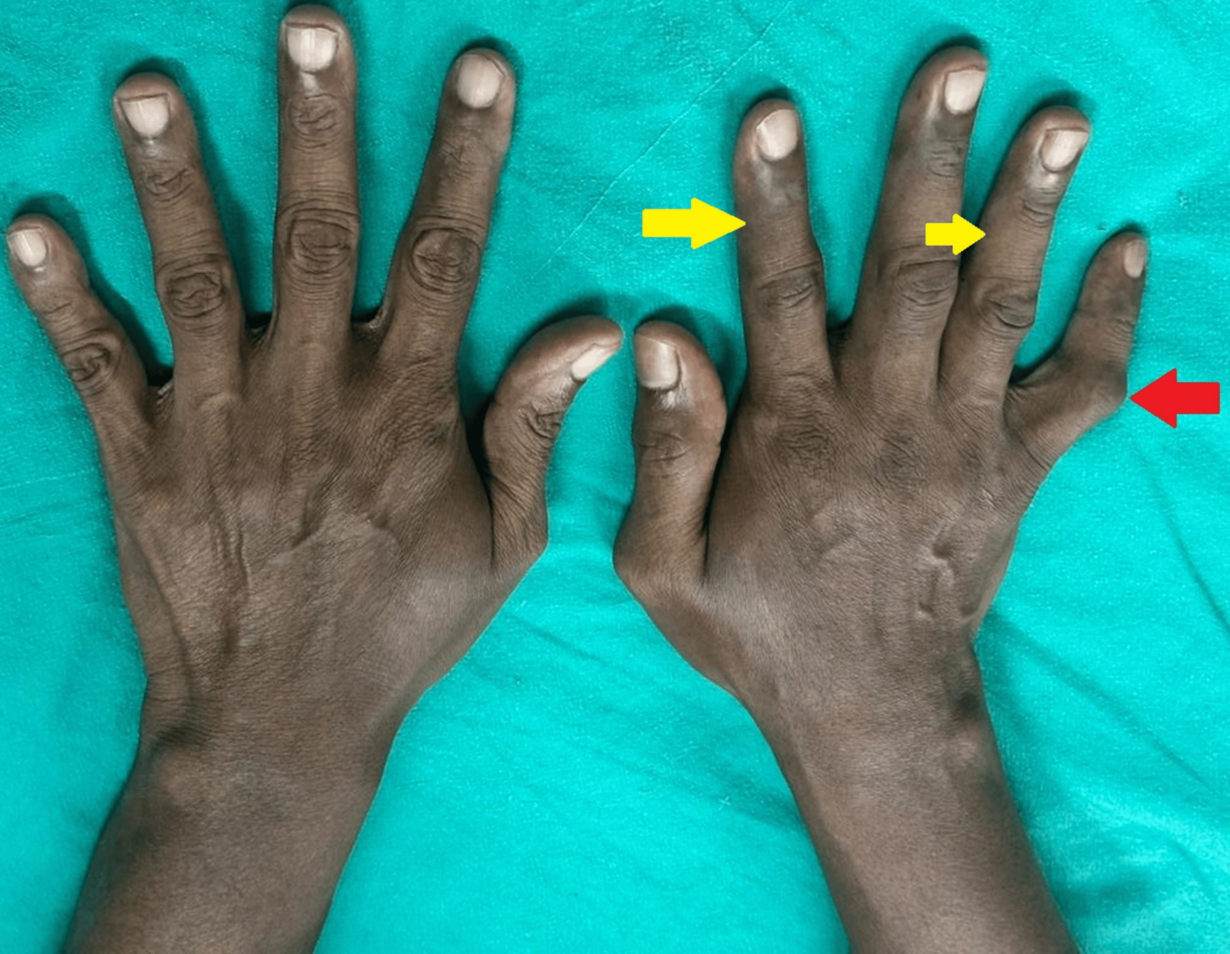
Postherpetic complex regional pain syndrome of right hand (after intervention) Decreased swelling (yellow arrows); persistent flexion deformity of right little finger (red arrow)

The median nerve block was performed at the carpal tunnel. Seven days after the intervention, the patient reported significant pain relief, and by day 15, pain remission was complete. His baseline VAS score was 7, and at 15 days, it was 0. The patient's range of motion improved, and the swelling decreased notably (Figure 3).

As the patient was right-handed, following the intervention, he was able to perform all the ADLs. According to the Sollerman Hand Function Test, his hand function improved from 'The task was completed, but with great difficulty, or the task was not carried out with the prescribed hand-grip, or the task was not completed within 40 seconds but within 60 seconds' to 'The task was carried out without any difficulty within 20 seconds and with the prescribed hand-grip of normal quality' [8]. Finally, the patient was able to return to work 20 days after the baseline visit (Barthel index: 100/100).

## Discussion

To the best of our knowledge, this is the first case report globally to describe comprehensive rehabilitation interventions for CRPS following a herpes zoster infection. It suggests that a holistic rehabilitation plan can be highly effective in improving both pain control and functional recovery [6,7,9].

Herpes zoster infection is an established risk factor for CRPS, especially when the outbreak involves the sensory nerves, as in this case, where the vesicular eruptions were localized to the ulnar distribution [10]. Research indicates that CRPS can follow various noxious insults, including trauma, surgery, and viral infections like herpes zoster [11]. It is believed that the inflammatory response triggered by the infection leads to peripheral sensitization and abnormal neuroplasticity, contributing to the pain and functional impairments characteristic of the condition [12,13]. In this patient, persistent pain, disability, and the development of a partial claw hand deformity - despite initial pharmacologic treatment with gabapentin, pregabalin, and occasional opioids - reflect the refractory nature of the syndrome.

A key factor in successful CRPS management is early intervention with a multidisciplinary approach [6,14]. The literature emphasizes the importance of combining pharmacological treatments with physical therapy and interventional pain management techniques, particularly for cases that do not respond to conventional analgesics [15]. Nerve blocks, such as those used in this case with methylprednisolone and lignocaine, are commonly employed in CRPS for both diagnostic and therapeutic purposes [16]. Studies show that sympathetic and motor nerve blocks can reduce pain and improve mobility by interrupting the aberrant nerve signaling that drives pain and dysfunction [17]. For instance, one study found that sympathetic blocks, especially when combined with physical therapy, significantly improved pain scores and functional outcomes for CRPS patients [18]. The success of the ulnar and median nerve blocks used in this case, with complete resolution of pain and a marked improvement in the range of motion, aligns with these findings.

The rehabilitation strategy used in this case involved progressive range-of-motion exercises and strengthening of the intrinsic hand muscles. This is another well-supported component of CRPS management. Research demonstrates that structured physical therapy aimed at restoring normal joint movement, improving muscle strength, and preventing further deformities plays a critical role in enhancing recovery and reducing disability [6]. In this patient, the gradual introduction of physical therapy after the nerve blocks contributed to the restoration of hand function and reduction of swelling, allowing a return to daily activities and work.

## Conclusions

This case highlights the importance of a multimodal approach for managing CRPS following a herpes zoster outbreak. Despite persistent severe pain and functional impairment, a combination of pharmacologic management and targeted physical rehabilitation proved effective. Nerve blocks, specifically targeting the ulnar and median nerves, provided significant pain relief and improved functional capacity. This case also underscores the importance of early, proactive rehabilitation in restoring hand function and alleviating disability, which is crucial for optimizing recovery in CRPS patients.

Additionally, it emphasizes the need for individualized, comprehensive treatment strategies that combine pain management with physical therapy to address both sensory and motor deficits associated with CRPS. This case serves as an important reminder that, with appropriate interventions, patients with complex pain syndromes can achieve significant functional improvement.
